# Fe^3+^-Substituted Sr_6_YAl(BO_3_)_6_ Red Pigments Designed Without Pb, Cd, or Hg: Colour Performance, Chemical Stability, and Elemental Release Screening

**DOI:** 10.3390/toxics14100914

**Published:** 2026-10-16

**Authors:** Jiajun Feng, Qimiao Huang, Junyuan Liang, Yuyuan Chen, Qiuhua Huang, Zihong Jiang, Peixuan Chen, Jian Kang, Xiang Li

**Affiliations:** New Energy and New Materials Research Center, School of Environmental and Chemical Engineering, Zhaoqing University, Zhaoqing 526061, China; 202424121249@stu.zqu.edu.cn (Q.H.);

**Keywords:** inorganic pigment, Fe^3+^ chromophore, borate, Pb/Cd/Hg replacement, chemical stability, ICP-OES, elemental release, safer-by-design

## Abstract

Red inorganic pigments traditionally rely on Pb-, Cd-, Hg-, or Cr(VI)-based colourants that carry avoidable hazard and exposure risks. Here, a family of Sr_6_YAl_1−x_Fe_x_(BO_3_)_6_ (0 ≤ x ≤ 1) pigments was prepared by solid-state reaction using Fe^3+^ as the chromophore and no Pb-, Cd-, Hg-, Cr(VI)-, or As-containing precursors. X-ray diffraction shows that the trigonal Sr_6_YAl(BO_3_)_6_-type framework (space group R-3) is retained as a single phase throughout the series; on the basis of isovalent charge, six-coordinate geometry and matched ionic radii, Fe^3+^ substitutes for octahedral Al^3+^ to form a continuous solid solution, and SEM-EDS mapping confirms a broadly homogeneous, single-phase Fe-containing borate. Increasing Fe content strengthens visible absorption and moves the colour from white to a light reddish-orange (warm tan), with the x = 1 end member giving L* = 69.69, a* = 10.08, b* = 12.79 and C* = 16.28. The colour is essentially unchanged in water and dilute acetic acid, shows a moderate shift in alkaline media (ΔE* = 8.05–10.21), and changes markedly only in 0.1 mol L^−1^ HCl (ΔE* = 15.07). Elemental release was screened by immersing 1.00 g of powder in 50 mL of water (H1), 0.01 mol L^−1^ acetic acid (H2) or 0.1 mol L^−1^ HCl (H3) for 24 h at room temperature, with each leachate read three times by Inductively Coupled Plasma Optical Emission Spectroscopy (ICP-OES). Water and dilute acid gave similar, modest release, whereas HCl mobilized substantial B, Sr and Y (530.72, 4639.42 and 470.54 mg L^−1^, i.e., up to 41.87%, 45.15% and 27.08% of the nominal inventory) but very little Fe (0.156 mg L^−1^, only ≈0.014% of the Fe inventory, and below detection in H1 and H2). The Fe chromophore is thus effectively immobilized even when the borate framework is attacked, decoupling colouration from chromophore leaching. The pigments demonstrate source-level elimination of regulated toxic metals together with strong chromophore retention; the non-standardized extraction provides a conservative release-potential screen rather than a regulatory compliance determination.

## 1. Introduction

Inorganic pigments offer thermal, photochemical and weathering stability that few organic colourants can match [1,2,3]. Vivid red formulations have long depended on red lead (Pb_3_O_4_), lead chromates (PbCrO_4_), cadmium sulfoselenides (CdS_1−x_Se_x_) and mercury sulfide (alpha-HgS, vermilion). Their colour performance is well established, yet the Pb, Cd, Hg and Cr(VI) they contain pose hazards throughout manufacture, service, recycling and disposal [4,5,6]. Removing such elements at the material-design stage is intrinsically safer than restricting exposure only after a product has entered use [7,8].

The challenge is not merely to delete a hazardous element, but to retain the visible absorption that produces an intense red [9]. Environmentally preferable pigments increasingly exploit crystal chemistry and chromophore coordination to tailor that absorption without conventional toxic metals [10,11]. Fe^3+^ is well suited to this role: it is abundant, inexpensive, non-regulated and redox-stable in air, with no higher-valent, mobile oxidation state analogous to Cr(VI), and it is readily accommodated in oxide lattices [12,13]. Fe-bearing perovskites and ferrites already provide red-to-brown shades together with useful near-infrared reflectance and high-temperature stability [14,15]. More broadly, coordination-controlled transition-metal colours such as YInMn blue demonstrate how a host lattice can intensify intrinsically weak d-d transitions [16,17]. Beyond pigment applications, octahedrally coordinated Fe^3+^ is also an established luminescent centre in oxide hosts: the ^4^T_1_(^4^G) to ^6^A_1_(^6^S) transition yields red-to-near-infrared emission in LiGa_5_O_8_, double-perovskite A_2_BB’O_6_, and Ca_2_MgTeO_6_ phosphors [18,19,20,21], and the Tanabe-Sugano diagram of the d^5^ configuration rationalizes the rich crystal-field dependence of its energy levels [22]. This dual identity-as a visible-light absorber and a potential emitter-underlines the importance of understanding Fe^3+^ coordination in any oxide host.

Borate lattices are particularly versatile hosts for both luminescent and pigmentary doping because they combine low phonon energy, wide optical transparency, good thermal stability, and relatively mild synthesis temperatures [23]. The hexaborate family A_6_MM’(BO_3_)_6_ (the STACK-type structure) has attracted sustained attention as a phosphor host: Ce^3+^-activated A_6_MM’(BO_3_)_6_ compounds show efficient blue emission [24], Tb^3+^- and Gd^3+^-doped Sr_6_YAl(BO_3_)_6_ exhibit characteristic f-f photoluminescence [25], and more recently Sr_6_LuAl(BO_3_)_6_:Dy^3+^ yellow and Sr_6_LuAl(BO_3_)_6_:Ce^3+^ blue phosphors have been developed for white-light LEDs [26,27,28]. Related borate systems such as Ba_3_Eu(BO_3_)_3_, Li_6_Lu(BO_3_)_3_:Ce^3+^, Tb^3+^, and Na_3_YB_8_O_15_:Sm^3+^/Eu^3+^ further demonstrate the capacity of borate frameworks to support intense rare-earth emission [29,30,31]. These studies collectively establish that the octahedral M’ site in Sr_6_YAl(BO_3_)_6_-type borates is chemically flexible and can accommodate a range of trivalent cations without structural collapse.

Fe^3+^ is one of the most promising chromophoric metal ions for inorganic pigments, owing to its environmentally friendly nature, non-toxicity to humans, and tunable colour in different materials. For example, γ-FeOOH exhibits colours ranging from yellow to dark yellow with good colour saturation [32]. (Fe,Cr)_2_O_3_ exhibits a black colour with high near-infrared reflectivity due to the combined effect of Fe^3+^ and Cr^3+^, showing potential as a replacement for carbon black [33]. It is possible to construct an “all-Fe^3+^ full-colour pigment system” by combining various Fe^3+^-based materials.

Sr_6_YAl(BO_3_)_6_ offers octahedral Al sites suited to Fe^3+^. The host crystallizes in the trigonal space group R-3 and is built from Sr-O polyhedra, Y- and Al-centred octahedra and planar BO_3_ groups [25]. Replacing Al^3+^ with Fe^3+^ is isovalent and therefore requires no charge compensation, and the six-coordinate Shannon radii are close-0.645 A for high-spin Fe^3+^ versus 0.535 A for Al^3+^, a mismatch of only about 21 percent-so a broad substitutional solid solution is crystallochemically plausible [34]. Unlike rare-earth activators, whose sharp f-f transitions are suited to narrow-band phosphors, Fe^3+^ produces broad, parity-forbidden d-d absorption bands in the visible region that are ideal for pigmentary colouration [35]. The same crystal-field splitting that governs Fe^3+^ luminescence therefore also controls its pigmentary hue, making the borate host a useful platform for correlating coordination environment with optical response.

A credible replacement must, however, be judged on more than colour. Bulk composition, chemical stability and transfer into a contacting solution answer distinct questions, and colour retention by itself is no guarantee of low dissolution; the two are best measured independently, as migration and bioaccessibility studies of toys and jewellery have shown for consumer articles [36,37]. Coupling source substitution with such release screening is consistent with the European safe-and-sustainable-by-design framework [38]. We therefore combine phase and optical characterization of Sr_6_YAl_1−x_Fe_x_(BO_3_)_6_ with chemical-exposure tests and Inductively Coupled Plasma Optical Emission Spectroscopy (ICP-OES), aiming to (i) establish how the Fe content governs the structure and colour, (ii) identify the media that produce the largest colour change, and (iii) quantify the release of the constituent elements under the stated screening conditions, and in particular to determine whether the Fe chromophore itself is retained.

## 2. Materials and Methods

### 2.1. Pigment Synthesis

Sr_6_YAl_1−x_Fe_x_(BO_3_)_6_ powders with nominal x = 0, 0.25, 0.50, 0.75, 0.80, 0.85, 0.90, 0.95, and 1.00 were prepared by a conventional solid-state route. SrCO_3_ (99.99%, Aladdin Reagent Co., Ltd., Shanghai, China), Y_2_O_3_ (99.99%, Aladdin Reagent Co., Ltd., Shanghai, China), Al_2_O_3_ (99.99%, Aladdin Reagent Co., Ltd., Shanghai, China), H_3_BO_3_ (analytical grade, Sinopharm Chemical Reagent Co., Ltd., Beijing, China), and Fe_2_O_3_ (99.99%, Aladdin Reagent Co., Ltd., Shanghai, China) were used as received. H_3_BO_3_ was added at 10 mol% excess to compensate for volatilization at high temperature.

The weighed reagents were ground in an agate mortar for 30 min, transferred to alumina crucibles, pre-calcined at 700 K for 5 h, and calcined at 1273 K for 4 h. Heating and cooling rates were 5 K min^−1^. After cooling, the products were ground again for 30 min before characterization.

### 2.2. Structural, Microstructural, and Optical Characterization

Powder X-ray diffraction patterns were collected on a Bruker D8 Advance diffractometer(Bruker AXS GmbH, Karlsruhe, Germany) using Cu Kα radiation (λ = 1.5406 Å) over 2θ = 10–80° at 5° min^−1^ and 15–120° at 4° min^−1^ for phase identification and Rietveld refinement, respectively. The crystal structures were refined using the Rietveld method with Z-Rietveld (Ver. 2.9.2.0 for Windows) software [39]. Morphology and element distributions were examined with a Thermo Scientific Apreo 2C field-emission scanning electron microscope (Thermo Fisher Scientific Inc., Waltham, MA, USA) equipped with an Oxford ULTIM Max65 energy-dispersive X-ray spectrometer (Oxford Instruments plc, Oxford, UK).

UV-visible diffuse reflectance spectra were recorded from 200 to 800 nm on a Shimadzu UV-2600 spectrophotometer(Shimadzu Corporation, Kyoto, Japan) using BaSO_4_ as the reference.

Colour coordinates were measured with a 3nh NR110 portable colourimeter (Shenzhen 3nh Technology Co., Ltd., Shenzhen, China) on powder samples in the CIE L*a*b* colour space using illuminant D65, the 10° standard observer, d/8 geometry, specular component included, and a Φ8 mm aperture. The instrument was calibrated with its standard white tile before each batch. Each sample was measured three times at different positions, and the mean was reported. Chroma, hue angle, and total colour difference were calculated as C* = [(a*)^2^ + (b*)^2^]^1/2^, *h*° = tan^−1^ (b*, a*), and ΔE* = [(ΔL*)^2^ + (Δa*)^2^ + (Δb*)^2^]^1/2^ [40].

### 2.3. Chemical Exposure Tests

The chemical stability of the optimal nominal x = 1.00 powder was evaluated by immersion in six media spanning neutral, acidic, and alkaline environments: distilled water, 0.1 mol L^−1^ HCl (strong acid), 0.01 and 0.05 mol L^−1^ CH_3_COOH (weak acid at two concentrations), 1 mol L^−1^ NaHCO_3_ (weak base), and 1 mol L^−1^ NaOH (strong base). For each medium, 1.00 g of powder was weighed into a 50 mL polypropylene (PP) centrifuge tube and contacted with 50 mL of the freshly prepared solution, giving a solid-to-liquid ratio of 1 g:50 mL (1:50, mass/volume). The sealed tubes were agitated end-over-end on a tube rotator at room temperature (22 ± 3 °C) for 24 h; each immersion condition was prepared as a single batch, and the recovered leachate was read three times on the instrument (reported as mean ± SD, Section 2.4); reagent blanks were carried through the procedure. After immersion, the tubes were centrifuged. An aliquot of the supernatant from three representative media—distilled water, 0.01 mol L^−1^ CH_3_COOH, and 0.1 mol L^−1^ HCl (denoted H1, H2, and H3)—was retained for the elemental release analysis described in Section 2.4, while the solid was recovered for the colour measurements. The recovered powder was washed three times with distilled water and finally once with anhydrous ethanol until the washings were neutral (pH ≈ 7), dried in a convection oven at 60 °C for 12 h, and gently re-ground in an agate mortar to restore a homogeneous powder. The CIE L*a*b* coordinates of each treated powder were then measured under exactly the same colorimetric conditions as the as-prepared sample, and the total colour difference relative to the untreated powder, ΔE*, was calculated from the expression given in Section 2.2.

### 2.4. ICP-OES Analysis

The three solutions designated H1, H2, and H3 were the 24 h leachates from the chemical-exposure test in Section 2.3 and corresponded to distilled water, 0.01 mol L^−1^ CH_3_COOH, and 0.1 mol L^−1^ HCl, respectively. These solutions were leachates rather than total digests; the results therefore quantify release from the powder under the stated conditions, not bulk elemental content. The supernatants were centrifuged, filtered through a 0.22 μm membrane, and acidified to 2% (*v*/*v*) with ultrapure HNO_3_ before analysis. Reagent blanks were carried through the procedure.

The leachates were analyzed for B, Sr, Y, and Fe by ICP-OES on a Thermo Scientific iCAP PRO instrument. Settings are summarized in Table 1: RF power, 1150 W; coolant, auxiliary, and nebulizer gas flows, 12.5, 0.5, and 0.5 L min^−1^, respectively; and an additional worksheet entry labelled ion gas, 15 L min^−1^. The quantitative emission lines were Sr 407.771 nm, B 249.678 nm, Fe 238.204 nm, and Y 360.073 nm. Quantification used an external calibration with an instrument blank and 1.0 and 5.0 mg L^−1^ standards. Calibration checks were recovered within 99.5–103.0%, and no certified reference material was analyzed. Samples above the calibration range were diluted with 2% (*v*/*v*) HNO_3_ using the element- and medium-specific factors f in Table 1; final concentration was calculated as *C* = *f* × *C*_0_, where *C*_0_ is the diluted instrument reading. The instrumental detection limit was reported as <0.01 mg L^−1^. For Fe and Y in H1 and H2, which were measured after tenfold dilution, results below the corresponding practical reporting limit of approximately 0.1 mg L^−1^ (approximately 5 mg kg^−1^ relative to the starting solid) are reported as not detected (ND).

Each leachate was read three times, and the results are expressed as the mean ± standard deviation of the three instrument readings. These are instrumental replicates of a single immersion extract, not independently prepared extraction replicates; the reported standard deviations therefore describe instrumental repeatability only. Because 1.00 g of powder was contacted with 50 mL of leachant (Section 2.3), the mass-normalized release was calculated as release (mg kg^−1^) = C (mg L^−1^) × 0.050 L/0.001 kg = 50C, and the fraction released relative to the nominal elemental inventory of Sr_6_YFe(BO_3_)_6_ was estimated from the formula mass.

## 3. Results and Discussion

### 3.1. Phase Formation and Microstructure

**Figure 1:**
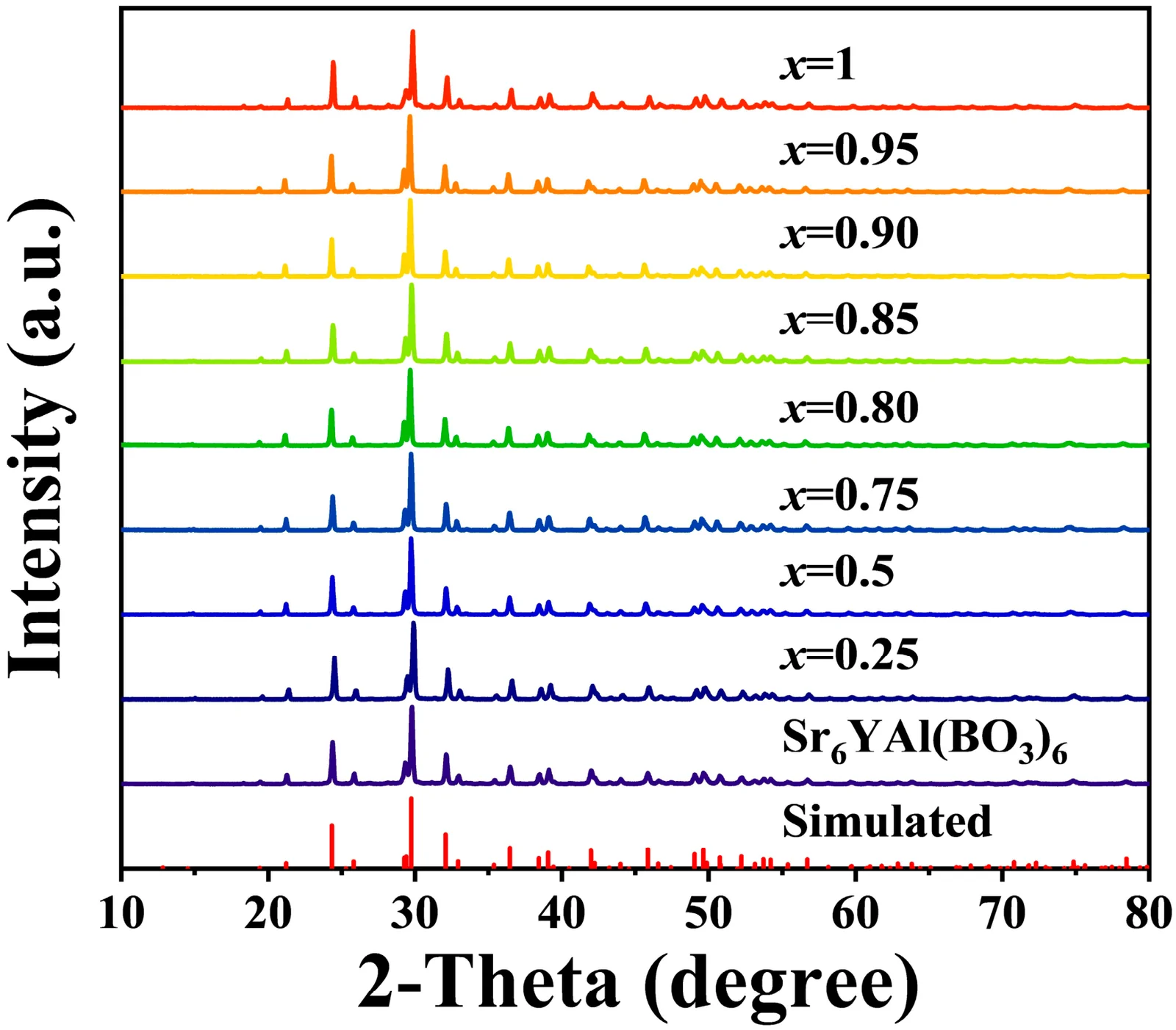
Powder XRD patterns of nominal Sr_6_YAl_1−x_Fe_x_(BO_3_)_6_ compositions (0 ≤ x ≤ 1), with the simulated Sr_6_YAl(BO_3_)_6_ host pattern shown for comparison.

The powder XRD patterns of the nominal Sr_6_YAl_1−x_Fe_x_(BO_3_)_6_ series (0 ≤ x ≤ 1) are compared with the simulated host pattern in Figure 1. For every composition, all principal reflections index to the trigonal Sr_6_YAl(BO_3_)_6_ host structure (space group R-3, No. 148) [24], with no detectable reflection from any crystalline secondary phase (such as α-Fe_2_O_3_, a borate, or a rare-earth oxide) and no peak splitting; the host-type pattern remained unchanged through to x = 1. Such single-phase, isostructural behaviour over the entire composition range establishes a continuous Sr_6_YAl_1−x_Fe_x_(BO_3_)_6_ solid solution, in direct analogy to the complete YAl_1−x_Fe_x_O_3_ solid solution (0 ≤ x ≤ 1), in which Fe^3+^ substitutes for octahedral Al^3+^ and the body colour evolves from yellow to red with increasing iron content.

The Fe-for-Al site assignment follows directly from the crystal chemistry of the structure. The host provides four cation sites that differ in coordination, charge, and size: an eight-coordinate Sr^2+^ site (r = 1.26 Å, CN8), a six-coordinate octahedral Y^3+^ site (r = 0.900 Å, CN6; Y–O ≈ 2.30 Å), the six-coordinate octahedral Al^3+^ site (r = 0.535 Å, CN6) in the [AlO_6_] unit, and a three-coordinate planar B^3+^ site (r = 0.11 Å, CN3) in the [BO_3_] triangle. The Al octahedron is thus the only site that matches Fe^3+^ simultaneously in charge, coordination number, and size. Occupation of the Sr^2+^ site would require compensating a one-charge mismatch, which is incompatible with the defect-free, single-phase patterns, whereas the isovalent Fe^3+^→Al^3+^ replacement needs no charge compensation and preserves electroneutrality; the much larger Y^3+^ octahedron (Y–O ≈ 2.30 Å) is likewise a poor geometric match for the short Fe^3+^–O bond. This preference of Fe^3+^ for the compact trivalent octahedral site rather than the larger rare-earth site is precisely the behaviour established for Fe^3+^ in YAlO_3_ and in Sr/Ba borates.

**Figure 2:**
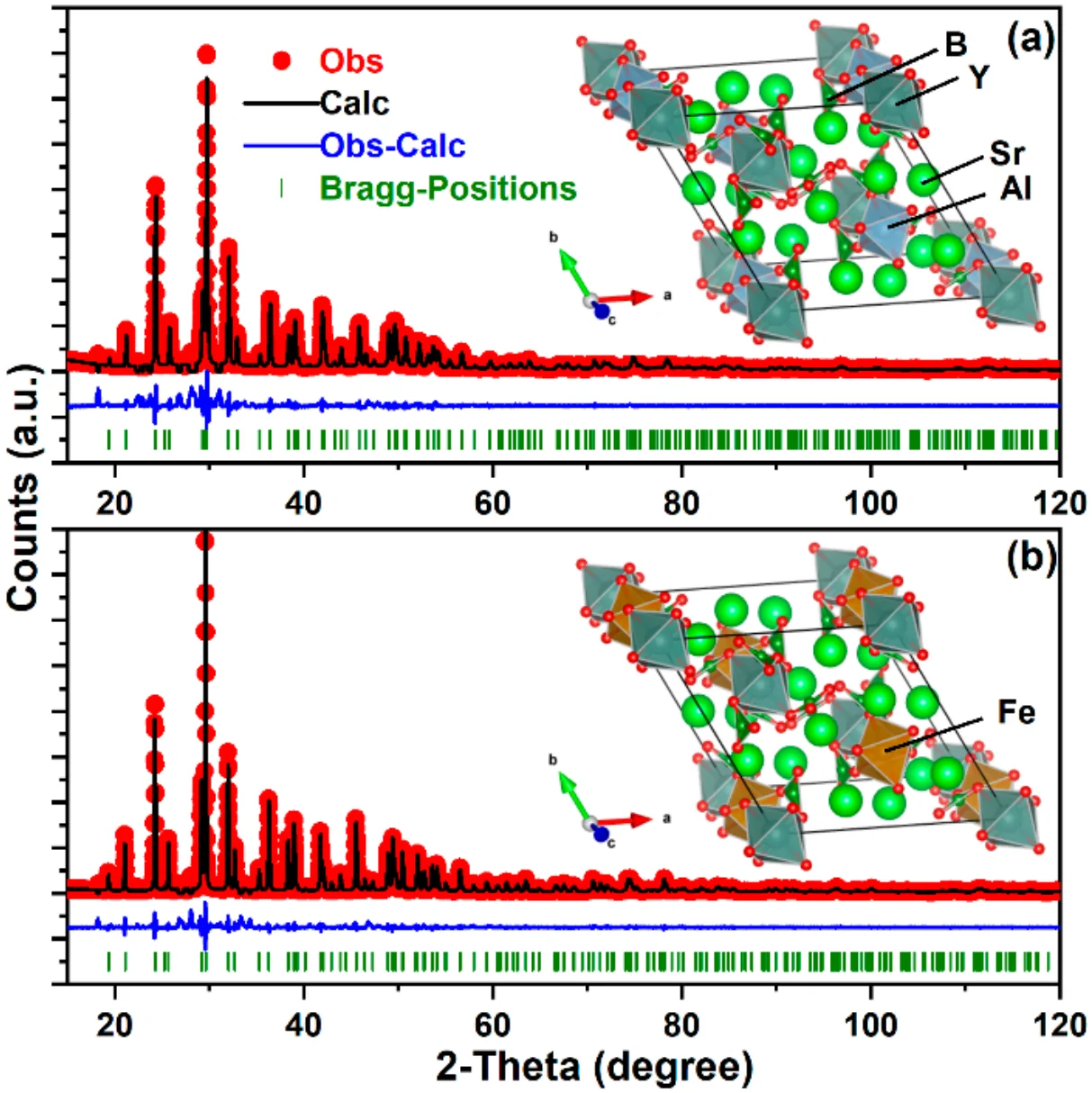
Rietveld refinement results and the corresponding crystal structures for (**a**) Sr_6_YAl(BO_3_)_6_ (*x* = 0) and (**b**) Sr_6_YFe(BO_3_)_6_ (*x* = 1).

The above statement is supported by the Rietveld refinement (Figure 2, Table 2 and Table 3) for Sr_6_YAl(BO_3_)_6_ (*x* = 0) and Sr_6_YFe(BO_3_)_6_ (*x* = 1). The substitution of Al^3+^ by Fe^3+^ not only increased the cell parameters but also increased the average bond length of the [(Al/Fe)O_6_] octahedra from 1.9176 Å (*x* = 0) to 2.0486 Å (*x* = 1), while the change in the [YO_6_] octahedra is insignificant, from 2.2991 Å (*x* = 0) to 2.2826 Å (*x* = 1).

**Figure 3:**
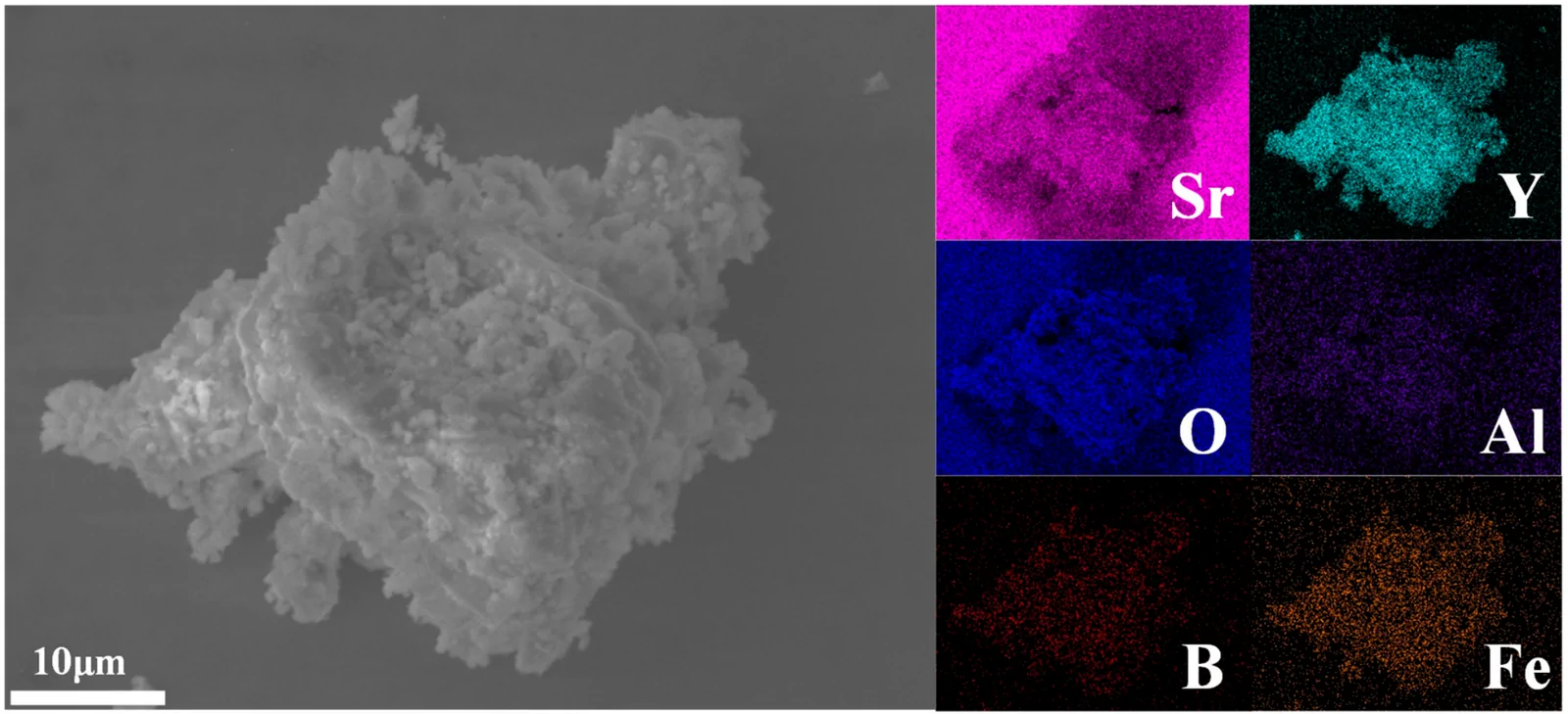
Representative secondary electron SEM image (10 μm scale bar) and EDS elemental maps (Sr, Y, Al, B, O, Fe) of the x = 0.9 composition, Sr_6_YAl_0.1_Fe_0.9_(BO_3_)_6_.

Figure 3 shows a representative secondary electron SEM image and the corresponding EDS elemental maps of the x = 0.9 composition, Sr_6_YAl_0.1_Fe_0.9_(BO_3_)_6_. As indicated by the 10 μm scale bar, the powder consists of irregular, compact agglomerates several tens of micrometres across (roughly 20–35 μm), each assembled from closely packed, submicrometre primary particles (about 0.5–1.5 μm). The primary particles are well crystallized and intimately fused, with no smooth amorphous skin, glassy binder phase, or abnormally large grains, consistent with well-developed crystallites and with the sharp, single-phase reflections in Figure 1; the mildly rough and porous appearance arises from inter-particle voids within the agglomerates rather than from intra-particle porosity.

Sr_6_YFe(BO_3_)_6_ is the deepest red among the samples but contains no Al^3+^. In contrast, Sr_6_YAl_0.1_Fe_0.9_(BO_3_)_6_ has a detectable Al^3+^ level (10% of the site), so it was chosen for EDS mapping to show the distribution of all elements. EDS mapping shows that Sr, Y, Al, B, O, and Fe are all distributed over the same region as the imaged agglomerate and closely follow its outline, with no element enriched at the surface, at grain boundaries, or around internal voids. In particular, the Fe map closely parallels the Al map across the entire particle, and neither Fe-rich clusters and Fe-depleted cores nor discrete iron-rich secondary particles are observed at the displayed scale. Such point-by-point Fe/Al co-localization, together with the uniform distribution of all framework elements, demonstrates at the microscale that Fe is incorporated homogeneously into the Al-containing framework of Sr_6_YAl_0.1_Fe_0.9_(BO_3_)_6_ rather than admixed as a separate iron-rich phase, which accounts for the uniform body colour of the powder. A weak residual Al signal is still detected, consistent with the 10 mol% Al remaining at this composition. The dense, elementally homogeneous agglomerates are also favourable for application, because minimizing surface segregation and exposed soluble phases is expected to limit ion release during aqueous and acidic exposure.

### 3.2. Optical Response and Colour

**Figure 4:**
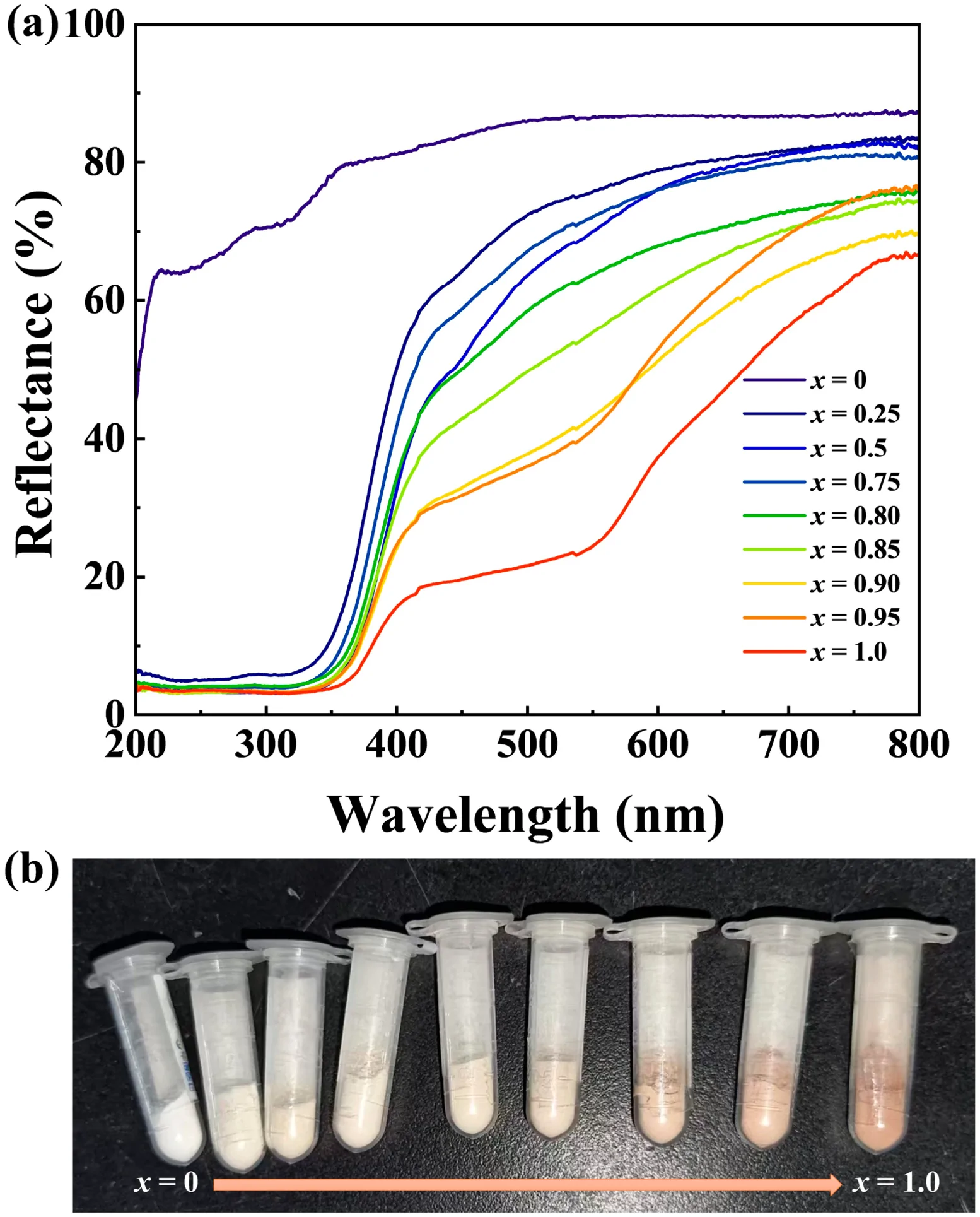
(**a**) UV-visible diffuse reflectance spectra of the nominal compositions. (**b**) Photograph of powders arranged in nominal synthesis order from *x* = 0 to *x* = 1. The photograph is qualitative because illumination and white balance were.

The UV-visible diffuse reflectance spectra of the series are shown in Figure 4a. The undoped Sr_6_YAl(BO_3_)_6_ host is bright and highly reflective throughout the visible region, with its absorption edge confined to the UV (below about 380–400 nm), consistent with its white appearance and wide intrinsic gap. Introducing Fe^3+^ adds strong absorption in the near-UV-to-blue range, assigned to O^2−^→Fe^3+^ ligand-to-metal charge transfer (CT) superimposed on the weaker, spin- and parity-forbidden Fe^3+^ (3d^5^) d–d transitions [41]. As x increases, this absorption intensifies and its low-energy edge shifts systematically to longer wavelengths, progressively depressing reflectance across the blue–green region (roughly 400–580 nm), while the red/near-IR region above about 600 nm remains comparatively reflective. With green light absorbed and red light returned, the powders become progressively warmer in tone, changing from the white host through pale cream and pinkish-orange to a soft reddish-brown at x = 1 (Figure 4b).

With increasing Fe^3+^ concentration, the L* values decreased, consistent with the enhanced overall absorption due to Fe^3+^ (Table 4). The increasing a* values indicated an enhanced red component in the pigments. The b* values first increased and then decreased slightly, implying an enhanced yellow component. The slight drop in the yellow component might be due to the rapid increase in the red component. The CIE L*a*b* coordinates of the nominal Sr_6_YFe(BO_3_)_6_ end member are L* = 69.69, a* = 10.08, and b* = 12.79, giving C* = 16.28 and *h*° = 51.8°. The relatively high lightness (L* ≈ 70) together with the moderately positive a* and b* places the colour in the first quadrant of the a*–b* plane, i.e., a soft, light reddish-orange to reddish-brown (warm tan) carrying both red and yellow components, rather than a deeply saturated red; the modest chroma (C* ≈ 16) indicates a muted, earthy tone. Such a pale warm shade is characteristic of Fe^3+^-based pigments and is well suited to non-toxic coatings and ceramics where a high-chroma heavy metal red is not required. The photographs in Figure 4b document the qualitative white-to-warm-brown evolution with *x*; however, because the camera illumination and white balance were not calibrated, the CIE L*a*b* measurements are taken as the quantitative colour record.

The nominal *x* = 1 end member gave L* = 69.69, a* = 10.08, and b* = 12.79, corresponding to C* = 16.28 and a warm reddish-orange appearance (Table 4). The positive a* and b* values indicate simultaneous red and yellow components. Photographs of the series show the transition from a white host to progressively warmer Fe-containing powders (Figure 4). Because camera white balance and illumination were not calibrated, the photographs document appearance qualitatively; the CIE L*a*b* measurements provide the quantitative colour values.

### 3.3. Colour Stability After Chemical Exposure

The colour response depended strongly on the contacting medium (Table 5; ΔE* is referenced to the as-prepared powder). Distilled water left all coordinates unchanged within rounding (ΔE* ≈ 0), i.e., no perceptible change. The two dilute acetic acid media caused only mild, dose-dependent shifts (ΔE* = 4.35 and 6.91 at 0.01 and 0.05 mol L^−1^, respectively), dominated by a slight loss of lightness and redness (lower L* and a*) rather than by yellowing. The alkaline media gave moderate changes (ΔE* = 8.05 in 1 mol L^−1^ NaOH and 10.21 in 1 mol L^−1^ NaHCO_3_), expressed mainly as higher b* and C*—a shift toward yellower, more chromatic tones with little change in hue; the weak base NaHCO_3_ produced a marginally larger shift than the strong base NaOH, pointing to carbonate-specific surface effects rather than pH alone. By far the largest alteration occurred in 0.1 mol L^−1^ HCl, where L* fell (ΔL* ≈ −13.4, pronounced darkening) while b* and C* rose, giving ΔE* = 15.07. Overall, the ranking distilled water < dilute acetic acid < alkaline media < 0.1 mol L^−1^ HCl shows that the pigment is effectively colour-stable in neutral, weakly acidic, and alkaline media, with appreciable alteration only under a strong acid extreme (pH ≈ 1, 24 h) far outside ordinary exposure.

Colourimetry reports the net visual outcome but is not chemically specific: it cannot, by itself, distinguish dissolution from precipitation, surface roughening, or reconstruction. An unchanged colour therefore does not imply zero elemental release, and colour retention and chemical stability are not equivalent. The leachates were therefore analyzed independently by ICP-OES (Section 3.4), which also tests whether the visual stability reflects genuine retention of the Fe chromophore rather than overall chemical inertness of the framework.

### 3.4. ICP-OES Concentrations in H1–H3

**Figure 5:**
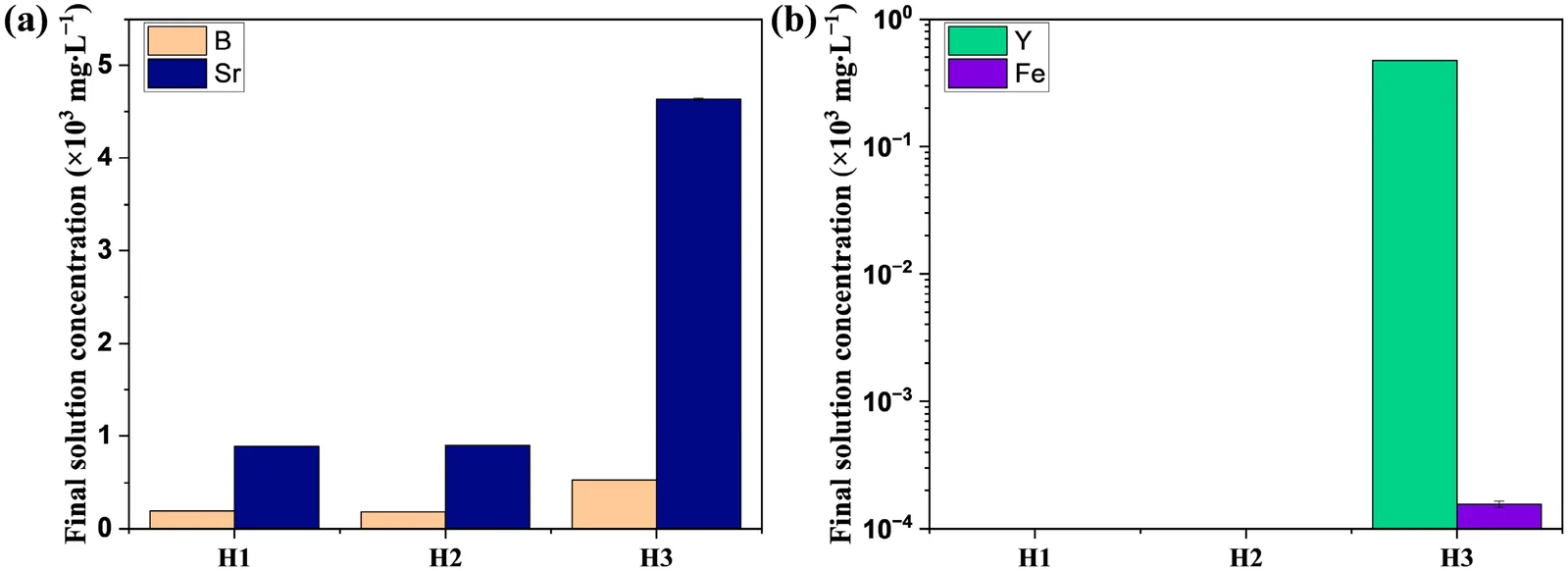
ICP-OES final solution concentrations for H1–H3 after dilution correction: (**a**) Sr and B; (**b**) Y and Fe on a logarithmic axis. Error bars are standard deviations of three instrument readings. ND values are labelled and are not plotted as numerical zeros.

The dilution-corrected concentrations—mean ± SD of three instrument readings on a single extract; ND = not detected at the stated practical reporting limit—are summarized in Table 6 and Figure 5. These leachate data answer a different question from the colour measurements: they quantify what leaves the solid, not what the solid looks like. The two are reported separately because colour retention and chemical stability are not equivalent. H1 (distilled water) and H2 (0.01 mol L^−1^ CH_3_COOH) were closely similar, with B = 186.36 and 177.96 mg L^−1^ and Sr = 891.05 and 901.05 mg L^−1^, respectively, and Y and Fe below detection in both. H3 (0.1 mol L^−1^ HCl) was far more aggressive, releasing B = 530.72, Sr = 4639.42, and Y = 470.54 mg L^−1^ (about 2.9× and 5.2× higher for B and Sr than in water, with Y rising from ND to high), whereas Fe remained very low at 0.156 mg L^−1^. The near-identical water and dilute-acid results show that mild acidity does not appreciably enhance attack; significant degradation is specifically triggered by strong acid [42].

With V = 50 mL and m = 1.00 g, the mass-normalized releases (mg kg^−1^ = 50 × C) and the fractions of the nominal elemental inventory are given in Table 7. B release was 8898–9318 mg kg^−1^ in water and dilute acetic acid (14.04–14.70% of inventory) and 26,536 mg kg^−1^ (41.87%) in HCl, while Sr release ranged from 44,553 mg kg^−1^ (8.67%) in water to 231,971 mg kg^−1^ (45.15%) in HCl; H3 additionally mobilized ≈27.08% of Y but only 0.014% of Fe (7.80 mg kg^−1^), while Fe and Y were below the ≈5 mg kg^−1^ reporting limit in H1 and H2. The decisive finding is that the Fe chromophore is essentially retained: even in the most aggressive medium, less than 0.02% of the Fe inventory is released, and Fe is undetectable in water and dilute acid. This decouples colour alteration from chromophore loss—the visible ΔE* in HCl arises mainly from framework dissolution and roughening (large Sr, B, and Y release) rather than from leaching of the Fe^3+^ colourant—which is consistent with the stable octahedral Fe site established above. In other words, colour retention reflects retention of the Fe chromophore, not overall chemical inertness of the framework. The two are decoupled: B, Sr, and Y can be mobilized while the Fe^3+^ colour centre remains in the solid.

For context, B and Sr numerically exceed the EN 71-3 Category I migration limits (1200 and 4500 mg kg^−1^) in all three screening media (B by 7.4–22.1× and Sr by 9.9–51.5×), whereas Y and Fe are not listed elements under EN 71-3. Two qualifications are essential. First, B and Sr are not the regulated toxic heavy metals of concern; the restricted elements Pb, Cd, Hg, Cr(VI), and As were neither used as precursors nor measured in this batch. Second, this 24 h, room-temperature, single-extract screen is not the standardized EN 71-3 extraction (0.07 mol L^−1^ HCl, 37 ± 2 °C, ≈2 h, protected from light), is based on one extraction with three instrument readings rather than independent replicates, and includes no certified reference material, so no formal compliance conclusion can be drawn; the small SDs quantify instrumental repeatability, not between-extraction reproducibility.

### 3.5. Safer-by-Design Implications and Limitations

From a hazard-reduction standpoint, the central achievement is elimination at the source. The pigment is synthesized without Pb-, Cd-, Hg-, or Cr(VI)-bearing precursors, and Fe^3+^—an abundant, low-toxicity, non-regulated ion—supplies the colour in place of a toxic metal chromophore, extending the metal-free design principle already demonstrated for other inorganic yellow–red pigments. Such substitution sits at the top of the safe-and-sustainable-by-design hierarchy of controls: removing a hazard at the design stage is intrinsically more effective than containing or treating a hazard that is never introduced, and it is the strategy explicitly prioritized in the European safe-and-sustainable-by-design framework. Because bulk composition and migration are distinct quantities—the former describes what the material contains and the latter what can transfer into a contacting phase—both were assessed here, since total content alone does not capture exposure, as documented for toys and jewellery [37].

The release data show that the two goals of safer-by-design are met together at the chromophore level. Even in the most aggressive medium (0.1 mol L^−1^ HCl), less than 0.02% of the Fe inventory is released and Fe is below detection in water and dilute acid, so the non-toxic colourant is effectively immobilized rather than exchanged into solution; the strong acid mass loss is dominated instead by framework B, Sr and Y, which are not regulated toxic elements. Safety therefore rests on two separate claims: source-level elimination of regulated toxic metals, and retention of the non-toxic Fe chromophore. Framework release of B and Sr under strong acid is a residual exposure-control need, not a failure of the substitution. For service environments in which even this framework release is to be minimized, established ceramic inclusion technology offers a direct route: occluding the chromophoric oxide within a dense, chemically inert zircon (ZrSiO_4_) shell, or vitrifying it into a glaze/glass phase, physically isolates the particles and is proven to confer both high-temperature and chemical resistance, as demonstrated for zircon-encapsulated hematite [43]. Applied to the present borate, such an inclusion barrier would retain the framework together with the already immobile Fe chromophore. A pigment that combines source-level absence of regulated toxic metals with negligible chromophore migration therefore embodies the safer-by-design objective of an inherently low-hazard, low-exposure material.

## 4. Conclusions

Nominal Sr_6_YAl_1−x_Fe_x_(BO_3_)_6_ pigments (0 ≤ x ≤ 1) were synthesized without Pb-, Cd-, Hg-, Cr(VI)-, or As-containing precursors, replacing conventional toxic metal chromophores with Fe^3+^. XRD shows that the trigonal Sr_6_YAl(BO_3_)_6_-type framework (space group R-3) is retained as a single phase across the entire series; on the basis of isovalent charge, six-coordinate geometry, matched ionic radii, and the established site preference of octahedral Fe^3+^ in analogous aluminates and borates, Fe^3+^ is assigned to the octahedral Al site to form a continuous solid solution. Increasing x strengthens visible absorption and moves the body colour from white through cream to a soft reddish-orange (warm tan). The colour is essentially unchanged in water and dilute acetic acid, moderately altered in alkaline media, and markedly changed only in 0.1 mol L^−1^ HCl (ΔE* = 15.07).

ICP-OES decouples the behaviour of the Fe chromophore from that of the Sr–B–Y framework. Water and dilute acetic acid give similar, modest B and Sr release, whereas 0.1 mol L^−1^ HCl sharply raises B, Sr, and Y release (up to 41.87%, 45.15%, and 27.08% of the nominal inventory, respectively), while Fe remains below detection in H1/H2 and reaches only 0.014% of the inventory in H3. The Fe colourant is therefore effectively retained even when the framework is attacked, and the observed colour change reflects framework dissolution and roughening rather than chromophore leaching. Colour retention is thus attributed to Fe retention rather than to overall chemical inertness; an unchanged colour does not imply zero elemental release. The material thus achieves source-level elimination of regulated toxic metals together with strong chromophore retention; the remaining issue is not toxic metal release but strong acid mobilization of the framework (B, Sr), identifying a residual exposure-control need rather than a failure of the substitution. Independent standardized migration tests (e.g., EN 71-3/ASTM F_963_) with procedural blanks, replicates, and certified reference materials, together with targeted total-digestion analysis of trace regulated elements, are required before any regulatory or “contaminant-free” claim; where harsh service is expected, ceramic barriers such as ZrSiO_4_ encapsulation or vitrification should be evaluated to turn an inherently non-toxic composition into a demonstrably low-release product.

## Figures and Tables

**Table 1:** ICP-OES conditions and dilution factors transcribed from the analytical worksheet.

Parameter	Reported Setting
Instrument	Thermo Scientific iCAP PRO
RF power	1150 W
Coolant/auxiliary/nebulizer gas	12.5/0.5/0.5 L min^−1^
Worksheet gas entry	Ion gas, 15 L min^−1^
Quantitative wavelengths	Sr 407.771; B 249.678; Fe 238.204; Y 360.073 nm
Dilution factor for Sr, H1/H2/H3	500/500/2500
Dilution factor for B, H1/H2/H3	100/100/500
Dilution factor for Y, H1/H2/H3	10/10/100
Dilution factor for Fe, H1/H2/H3	10/10/10

**Table 2:** Structural parameters of Sr_6_YAl(BO_3_)_6_ by Rietveld refinement.

Space Group: *R*-3 (No. 148)						
*a* = 12.1931(1), *b* = 12.1931(1), *c* = 9.10361(15), *α* = 90.00°, *β* = 90.00°, *γ* = 120.00°						
Site	Type	*x*	*y*	*z*	Occupancy	*B*_iso_ (Å^2^)
Sr	Sr	0.04183(11)	0.47179(10)	0.30515(12)	1.0	2.33(4)
Y	Y	0.000000	0.000000	0.000000	1.0	2.34(8)
Al	Al	0.000000	0.000000	0.500000	1.0	1.11(16)
B	B	0.0505(17)	0.1878(15)	0.2421(18)	1.0	2.10(30)
O1	O	−0.0043(7)	0.7380(11)	0.8076(7)	1.0	3.10(20)
O2	O	0.0386(7)	0.8723(7)	0.1517(8)	1.0	2.53(16)
O3	O	0.0392(7)	0.1482(7)	0.3876(9)	1.0	2.13(16)

The site occupancies were fixed in the refinement.

**Table 3:** Structural parameters of Sr_6_YFe(BO_3_)_6_ by Rietveld refinement.

Space Group: *R*-3 (No. 148)						
*a* = 12.22273(6), *b* = 12.22273(6), *c* = 9.1846(1), *α* = 90.00°, *β* = 90.00°, *γ* = 120.00°						
Site	Type	*x*	*y*	*z*	Occupancy	*B*_iso_ (Å^2^)
Sr	Sr	0.04220(9)	0.47098(9)	0.30663(11)	1.0	2.28(3)
Y	Y	0.000000	0.000000	0.000000	1.0	2.42(6)
Fe	Fe	0.000000	0.000000	0.500000	1.0	1.56(8)
B	B	0.0528(15)	0.1940(14)	0.232(2)	1.0	3.10(30)
O1	O	−0.0034(5)	0.7372(8)	0.8145(8)	1.0	2.71(17)
O2	O	0.0382(7)	0.8757(7)	0.1530(8)	1.0	2.62(13)
O3	O	0.0426(5)	0.1606(5)	0.3861(8)	1.0	1.96(15)

The site occupancies were fixed in the refinement.

**Table 4:** CIE L*a*b* coordinates of the Sr_6_YAl_1−x_Fe_x_(BO_3_)_6_ samples.

Sample	L*	a*	b*	C*	*h*°
*x* = 0.00	96.88	−0.33	3.06	3.08	96.16
*x* = 0.25	94.10	0.38	11.13	11.14	88.04
*x* = 0.50	91.52	1.02	14.59	14.63	86.00
*x* = 0.80	88.22	1.34	13.25	13.32	84.23
*x* = 0.85	86.87	1.07	16.44	16.47	86.28
*x* = 0.90	80.95	3.41	16.11	16.47	78.05
*x* = 0.95	73.18	8.39	14.99	17.18	60.76
*x* = 1.00	69.69	10.08	12.79	16.28	51.76

**Table 5:** CIE L*a*b* coordinates after 24 h exposure. The as-prepared powder is the ΔE* reference.

Treatment	L*	a*	b*	C*	ΔE*
As prepared	69.69	10.08	12.79	16.28	-
Distilled water	69.69	10.08	12.79	16.28	≈0
0.01 mol L^−1^ CH_3_COOH	67.03	6.70	12.13	13.86	4.35
0.05 mol L^−1^ CH_3_COOH	63.30	8.22	14.66	16.81	6.91
1 mol L^−1^ NaOH	67.24	12.99	19.89	23.76	8.05
1 mol L^−1^ NaHCO_3_	64.84	12.13	21.54	24.72	10.21
0.1 mol L^−1^ HCl	56.27	11.37	19.52	22.59	15.07

**Table 6:** Dilution-corrected ICP-OES solution concentrations. Values are mean ± SD of three instrument readings from one extraction. ND = not detected at the stated practical reporting limit.

Solution	B (mg L^−1^)	Sr (mg L^−1^)	Y (mg L^−1^)	Fe (mg L^−1^)
H1	186.36 ± 1.41	891.05 ± 0.92	ND	ND
H2	177.96 ± 0.85	901.05 ± 0.23	ND	ND
H3	530.72 ± 1.16	4639.42 ± 10.65	470.54 ± 0.62	0.156 ± 0.009

**Table 7:** Mass-normalized release and estimated fraction of the nominal elemental inventory of Sr_6_YFe(BO_3_)_6_. Values derive from one extraction and three instrument readings; EN 71-3 values are contextual benchmarks only.

Solution	B, mg kg^−1^ (% Inventory)	Sr, mg kg^−1^ (% Inventory)	Y, mg kg^−1^ (% Inventory)	Fe, mg kg^−1^ (% Inventory)
H1	9318 ± 71 (14.70%)	44,553 ± 46 (8.67%)	<5 (<0.006%)	<5 (<0.009%)
H2	8898 ± 43 (14.04%)	45,053 ± 12 (8.77%)	<5 (<0.006%)	<5 (<0.009%)
H3	26,536 ± 58 (41.87%)	231,971 ± 533 (45.15%)	23,527 ± 31 (27.08%)	7.80 ± 0.45 (0.014%)
EN 71-3 Category I	1200	4500	Not listed	Not listed

## Data Availability

Data will be made available upon request from the corresponding authors.
